# The Micrococci of Tuberculosis

**Published:** 1882-01

**Authors:** 


### THE MICROCOCCI OF TUBERCULOSIS.

Certain investigators have thought they had discovered the active agent in tuberculosis to be a micrococcal organism. Their experiments have been repeated by Dr. Deutschmann. The results which he has obtained do not confirm this view. For instance, some liquid from the anterior chamber of a child suffering from tubercles of the iris was injected into the eye of a rabbit, but without result, although it was found to contain numerous mobile micrococci. These results, Deutschmann concludes, can only be explained on three hypotheses:—

1. That micrococci have nothing to do with the tubercular infection, or with the tuberculous virus.

2. The micrococci serve only as the vehicles of the tubercular infection when they can germinate in a suitable soil, such as is furnished by the lower layer of the tubercular pus.

3. The specific organisms were placed in such unfavorable conditions that they had no pathogenetic action. It is conceivable, for instance, that the micro-organisms were so rapidly rendered harmless in the blood, that they had no effect, whereas the slower absorption of the lower pus layer gave them time to multiply in it.—*Med. and Surg. Reporter.*

